# An Investigation into Laser-Assisted Electrochemical Discharge Machining of Transparent Insulating Hard-Brittle Material

**DOI:** 10.3390/mi12010022

**Published:** 2020-12-27

**Authors:** Douyan Zhao, Zhaoyang Zhang, Hao Zhu, Zenghui Cao, Kun Xu

**Affiliations:** 1 Laser Technology Institute, School of Mechanical Engineering, Jiangsu University, Zhenjiang 212013, China; 2211803033@stmail.ujs.edu.cn (D.Z.); haozhu@ujs.edu.cn (H.Z.); xukun@ujs.edu.cn (K.X.); 2 Fengjiang Intelligent Technology Co., Ltd., Changzhou 213100, China; 2211803060@stmail.ujs.edu.cn

**Keywords:** electrochemical discharge machining, laser machining, glass, micro-groove

## Abstract

Electrochemical discharge machining (ECDM) and laser machining are emerging nontraditional machining technologies suitable for micro-processing of insulating and hard-brittle materials typified by glass. However, poor machinability of glass is a major constraint, which remains to be solved. For the micro-grooves processed by ECDM, the bottom surface is usually uneven and associated with protrusion structures, while the edges are not straight with obvious wave-shaped heat-affected zones (HAZs) and over-cutting. Besides, the cross section of the micro-grooves processed by the laser is V-shape with a large taper. To solve these problems, this study proposed the laser-assisted ECDM for glass micro-grooving, which combines ECDM and laser machining. This study compared morphological features of the single processing method and the hybrid processing method. The results show that ECDM caused cylindrical protrusions at the bottom of the microgrooves. After processing these micro-grooves by laser, the cylindrical protrusions were removed. However, the edge quality of the microgrooves was still poor. Therefore, we used the laser to get microgrooves first, so we got micro-grooves with better edge quality. Then we use ECDM to improve the taper of microgrooves and the cross-sectional shape of the microgrooves transformed from a V-shape to a U-shape.

## 1. Introduction

Insulating and hard-brittle materials typified by glass are used widely in many fields such as chemical engineering, electronics, communications, instrumentation, and nuclear engineering. Especially in some complex, harsh, or extreme working environments, the parts made of glass materials show excellent corrosion resistance and high temperature stability. Although glass possesses many excellent characteristics, these materials are typically hard and brittle, and are therefore difficult to machine by conventional approaches, which affects the machining quality and limits its further application [1].

Traditional methods that have been reported for glass machining, including diamond grooving, mechanical grinding, micro-abrasive air jet [2] and water jet [3], in which material removed because of brittle crack formation caused by the cutting tool squeeze or jet impingement. Defects such as cracks and chipping easily produced during processing, leading to poor processing accuracy and surface quality.

For higher machining efficiency, better processing accuracy and surface morphology, nontraditional technologies have proposed for glass in recent years [4], and the typical methods are laser machining [5,6,7] and electrochemical discharge machining (ECDM) [8,9,10,11,12], both of which are noncontact methods and removing materials in a stress-less manner. Therefore, noncontact methods can avoid the stress damage, and reduce the chipping and cracks [13] significantly improving the processing accuracy and surface quality.

Specifically, the two nontraditional methods have their own characteristics. For laser machining, the light beam focuses on a small area and the pulse energy emits within a short or ultrashort pulse duration, and therefore achieves extremely high energy intensity up to GW/cm^2^ even PW/cm^2^, leading to unique nonlinear light, heat, and force effects [14,15]. In the process of ultrashort laser processing, such as picosecond laser, the laser energy first absorbed by electrons in material, and the amount determined by the absorption rate of the material itself. The electrons in the material absorb photons and then migrate, and, then, the electrons excited to the conduction band further improve the energy absorption of the workpiece. When the electron density of the conduction band exceeds the critical value, a Coulomb explosion may occur, and helps increase material removal.

**Figure 1:**
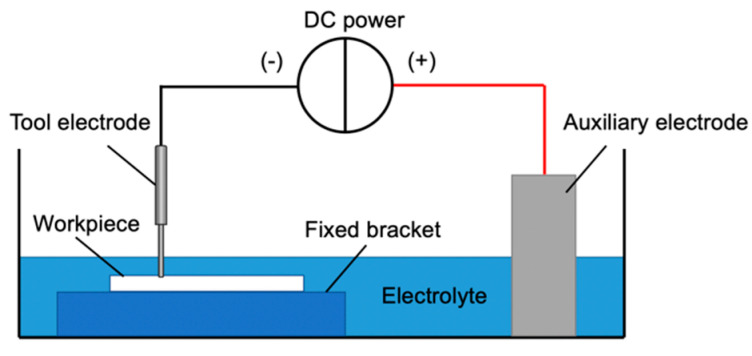
Schematic of the electrochemical discharge machining (ECDM).

The ECDM shown in Figure 1, where the tool electrode and auxiliary electrode immersed in the solution. After the DC power turned on, a current loop formed. As the reaction continues, the bubbles would fuse into a gas film, insulating the tool electrode from the working fluid. When the potential difference between the tool electrode and the working fluid increased up to the discharge voltage that could cause breakdown in the gas film, a spark discharge occurred, releasing a huge amount of energy. The emitted discharge energy led to high temperature and high pressure within the tiny distance between the tool electrode and material, and therefore the material may melt or even vaporize, and is then thrown away by high-pressure impact. Meanwhile, the rise of temperature also promoted the chemical corrosion of the alkaline solution on the workpiece. The two work together to increase material removal [16,17,18].

In the existing literature, scholars from various countries have carried out research on the micro-machining technologies of insulating and hard-brittle materials, and a wide range of hybrid processing methods have been proposed.

A hybrid method of ECDM and micro grinding using polycrystalline diamond (PCD) tools explored by Xuan et al. [19], which had experimentally shown the grinding under PCD tools reduced the surface roughness of ECDM structures. The fundamental material removal mechanisms of hard-brittle materials under indentation and material removal mechanisms in the gap between the tool side and the hole wall have been studied by Nath et al. [20]. Ho et al. [21] explored the feasibility of ECDM with an aiding flow jet on nonconductive quartz glass materials. This research found that an aid nozzle improved the drilling depth by ensuring that an enough flow of electrolyte was available for spark generation and debris removal. Chen et al. [22] added a magnetic field to ECDM. The mechanism of the magnetohydrodynamic effect in ECDM was researched. By using this hybrid method, the ECDM micro-hole drilling performance was significantly improved.

However, much work is still in early exploration and the experimental research stage. The hybrid processing technology for insulating and hard-brittle materials is still based on one of laser or spark discharge, and then introduces rotation, vibration, inflation, or grinding for aid. However, there is still little research on etching glass by combining the two main energies of laser and spark discharge to improve the processing quality. Therefore, there are many theoretical and technical issues that need to be studied for the optoelectronic composite stepwise processing of glass.

This study focused on the step-by-step composite processing of glass using different sequences of laser and ECDM. According to the thermal effect of laser shock wave and the principle of ECDM effect, this study compared and analyzed the morphology characteristics of different machining methods. It also analyzed the influence of laser pre-processing on forming an ECDM gas film and the ablation of materials.

## 2. Experimental Design

### 2.1. Experimental Setup

**Figure 2:**
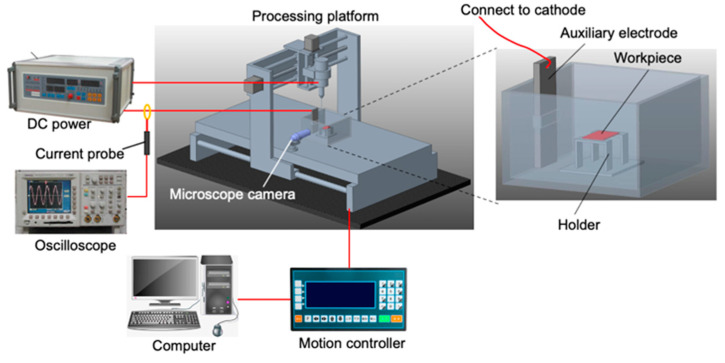
Schematic diagram of the experimental apparatus of ECDM.

As shown in Figure 2, an ECDM system has developed for this study, including pulse power supply (GKPT series, Shenzhen Shicheng Electronic Technology Co., Ltd. Shenzhen, China), current probe (CP8030B, Shenzhen Zhiyong Electronics Co., Ltd. Shenzhen, China), motion controller, four-axis linkage processing platform, microscope camera, tool electrode, auxiliary electrode, reaction tank, oscilloscope (TDS2012C, American Tek company, Beaverton, OR, USA), etc.

The solution tank (size—160 × 120 × 80 mm^3^) made of acrylic sheet fixed on the processing platform, which can precisely move along X–Y axes via a computer-controlled motion controller unit. The tool electrode clamped onto a vertical chuck rotated by a motor, and was moved up and down by the main spin. The negative pole of the DC pulse power supply connected to the tool electrode, and the positive pole connected to the auxiliary electrode. The high frequency current probe employed to monitor the current during ECDM, and the oscilloscope connected to it was used to display and store the collected current signal.

Tool electrodes and auxiliary electrodes are important in ECDM [23,24,25], and the material choice for them should be carefully considered. The tool electrode should not only have good electrical conductivity, but also needs to withstand the high temperature of electric spark discharge and strong alkali corrosion. Therefore, tungsten carbide (WC) was selected to make the tool electrode for this study. In addition, since the spiral thread electrode is more effective in expelling machining waste and renewing the electrolyte near the machining position compared to the smooth electrode, this experiment used a cylindrical tool electrode with a thread on the sidewall. The auxiliary electrode was made of block graphite with strong alkali resistance and good conductivity (size—80 × 20 × 15 mm^3^).

**Figure 3:**
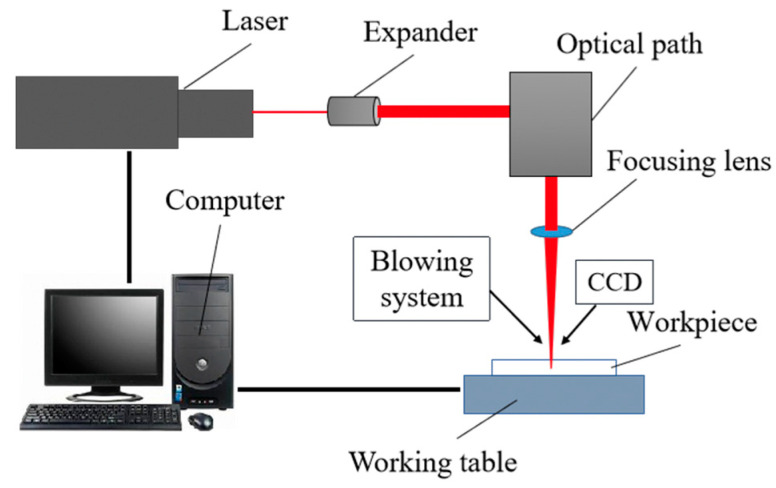
Schematic of picosecond laser machining system.

Figure 3 shows the schematic of picosecond laser machining system, which was mainly composed of a picosecond laser, computer control system, optical path system, four-axes precision motion system, optical measurement system (charge coupled device camera, (CCD camera)), and other auxiliary equipment. A Nd: YVO4 laser (Edgewave PX100-1-GM) was employed in this study, which was operating in Gaussian mode at 1064 nm wavelength with a 12 ps pulse duration and the beam quality is M2 < 1.3. The maximum output power was about 70 W, while the pulse repetition frequency can change from 0.2 to 1 MHz, resulting in a maximum pulse energy of up to 260 μJ at the frequency of 0.2 MHz. The output power was adjusted by changing the high voltage (HV) level working on the HV modulator, which is superior to controlling the pump current as the laser spot size not affected by the varied HV level in this laser machine. In addition, higher HV level results in more energy emission and therefore the increased laser intensity. The laser beam emitting from the generator first expanded by a beam expander (Eoptics VE-532–1064, JENOPTIK, Jena, Germany), then directed to a galvanometer (IntelliSCAN 14–1064, SCANLAB, Munchen, Germany) with a focal length of 100 mm and a typical marking speed of 2 m/s, and finally arrived at the target specimen surface. A mechanical beam blocker also included in the light path for a protection purpose. To keep the laser system in a stable operating stage, deionized and filtered water was looping inside the laser machine to remove the generated heat and hence maintain a constant temperature. By using this optical setup, a focusing laser spot of ∼20 μm in diameter achieved in the focal plane.

### 2.2. Machining Procedures

In this experimental study, the laser machining and ECDM was first carried out separately. In the ECDM experiment, the workpiece was a square piece of quartz with a size of 20 × 20 × 1 mm^3^, which was fixed to the bottom of the solution tank by a bracket. The electrolyte was a 30 wt.% NaOH solution, and the electrolyte level was adjusted to 2 mm above the workpiece. The ECDM parameters selected in this experiment are shown in Table 1. In the experiment, the layer was fed 3 times, and each feeding amount was 0.2 mm.

In this ultrashort pulsed laser micromachining process, the laser used to heat the material may cause melting, vaporization, plasma formation, or direct phase explosion as in the femtosecond laser. The workpiece with a size of 20 × 20 × 1 mm^3^ was fixed to the worktable of the laser system, and the CCD camera that comes with the laser, which is used for accurate focusing, such that the laser focused on the upper surface of the workpiece to process microgrooves. In the experiment, the scanning path designed by the computer in the laser processing system was a parallel line, the scanning distance between two adjacent parallel lines was 10 μm, the number of scanning lines was 20, and the scanning length was 2 mm. After each time the laser scanned a layer, the laser focus fed down by 20 μm to scan again, for 10 feeds. Because the laser focus diameter was 20 μm, the scanning distance between two adjacent parallel lines was 10 μm, and the scanning lines were 20, microgrooves with a width of around 200 μm were processed by this method. The laser machining parameters selected in this experiment are shown in Table 2. The scanning velocity and repetition times can be set by controlling the galvanometer. After the laser machining was completed, the workpiece was immersed in an alcohol solution for ultrasonic cleaning for 10 min and dried to remove the attached processing products.

**Figure 4:**
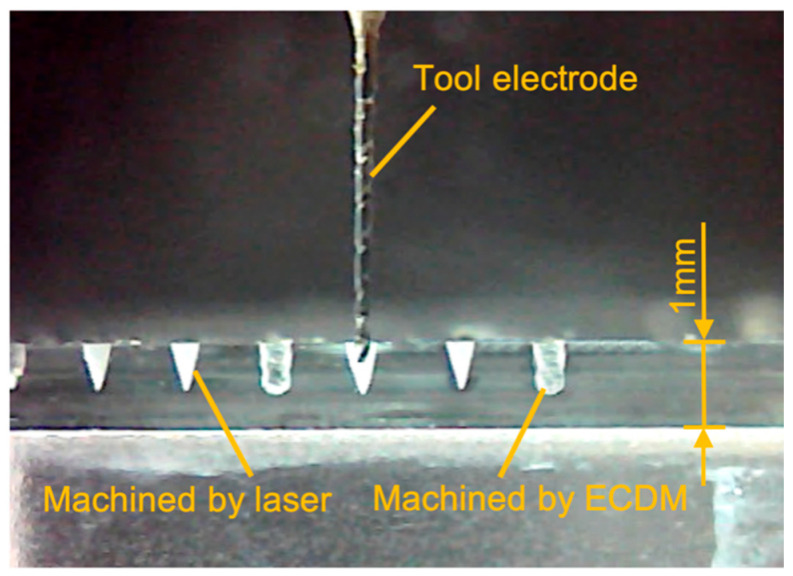
Cross-sectional morphology of the microgrooves respectively processed by laser and ECDM.

Figure 4 was taken by a microscopic camera, showing the cross-sectional morphology of the microgrooves, respectively processed by laser and ECDM. The results show that although the edge profile of microgrooves processed by laser is relatively flat and neat, the cross-sectional taper is relatively large, showing a clear V-shape. On the contrary, the microgrooves processed by ECDM have almost no taper, but the edges have obvious wave-shaped heat-affected zones.

In order to obtain microgrooves with a small taper and good surface quality, the laser machining and ECDM carried out in a certain sequence on the same position to form a hybrid machining method, which combined the advantages of the two methods.

**Figure 5:**
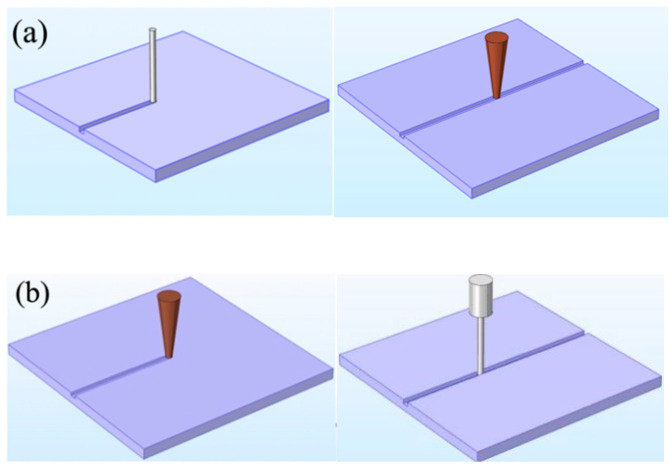
Processing diagram: (**a**) laser pre-groove processing and (**b**) ECDM pre-groove processing.

Figure 5 illustrates the processing sequence. Figure 5a shows ECDM pre-groove processing. The first step is to process the microgrooves on the workpiece by ECDM. Then the workpiece immersed in alcohol for ultrasonic cleaning for 10 min and dried to remove the attached processing products. The second step is to fix the workpiece to the laser processing platform. Then, use the CCD camera to observe the position of the microgroove, and use the software in the computer to focus on the center of a microgroove. In this way, the microgrooves processed by laser can completely overlap with the microgrooves processed by ECDM in the first step. The parameters used in these two steps were the same as described above. Except that the number of layer feeds in this ECDM experiment was one and the rotating speed is 0 RPM. Figure 5b shows laser pre-groove processing. The order of this processing method was reverse to that in Figure 5a. In the first step, a laser used to process microgrooves on the workpiece. Then the workpiece is immersed in alcohol for ultrasonic cleaning for 10 min and dried to remove the attached processing products. The second step is to fix it on the ECDM platform. Then observe the position of the microgroove with a microscopic camera, align the tool electrode at the center of a microgroove, and perform the ECDM. The parameters used in these two steps were the same as described above. Except that the rotating speed is 0 RPM.

After processing, the glass was cleaned and dried. The scanning electron microscope (SEM, Hitachi S-3400N) was used to observe the surface morphology of the processed microgrooves. Because the glass itself is an insulator, it is necessary to spray a metal film or carbon film on the material to form a conductive layer before observation.

## 3. Results and Discussion

### 3.1. ECDM Preprocess

**Figure 6:**
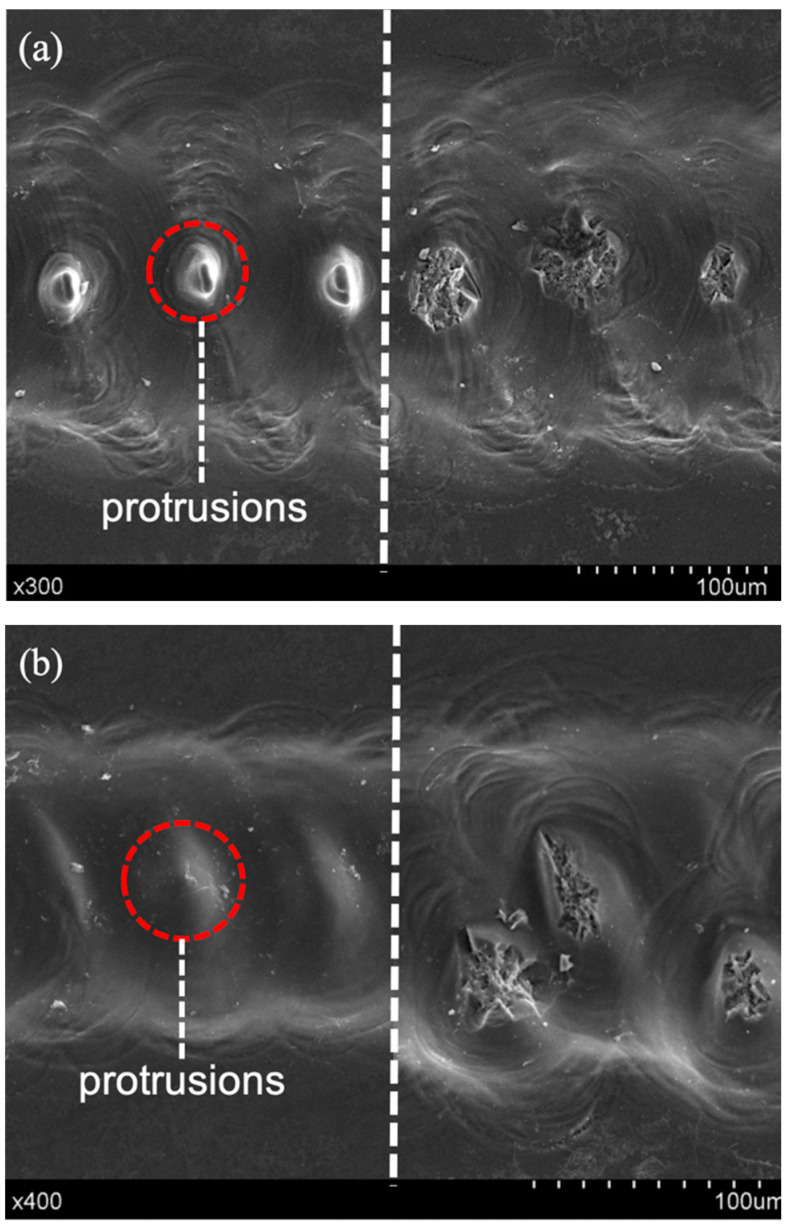
Morphology comparison of micro-grooves machined by ECDM only (left part) and hybrid method with laser processing. (**a**) 1000 Hz and (**b**) 800 Hz.

Figure 6 is the SEM image of a microgroove processed by ECDM. Taking the dotted line as the boundary in the figure, the left side shows the morphology of a microgroove produced by ECDM only, while the right side matches the result of post-processed by laser machining after ECDM. The ECDM parameters of Figure 6a were 1000 Hz and 0 RPM. Protrusions appear regularly at the bottom of the microgroove, and these protrusions are cylindrical with steep sidewalls. Further, the distance between the peaks of two adjacent protrusions is about 70 μm, while the bottoms not connected. Figure 6b shows the micro-grooves processed with different ECDM parameters (800 Hz and 0 RPM). Compared with Figure 6a, the regularity of the protrusions’ distribution decreases. The protrusions are nearly cone-shaped with a gentle sidewall. The distance between the tops of the adjacent two protrusions is about 30–80 μm, and the bottoms are connected.

**Figure 7:**
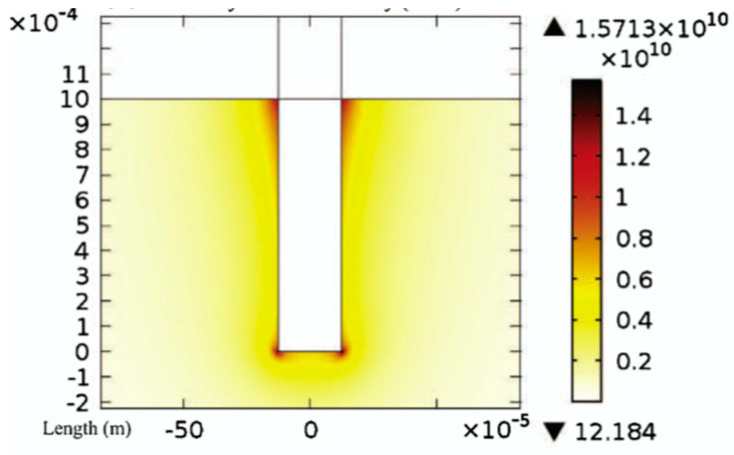
Finite element simulation of current density in electrochemical reaction on cylindrical electrode [26].

**Figure 8:**
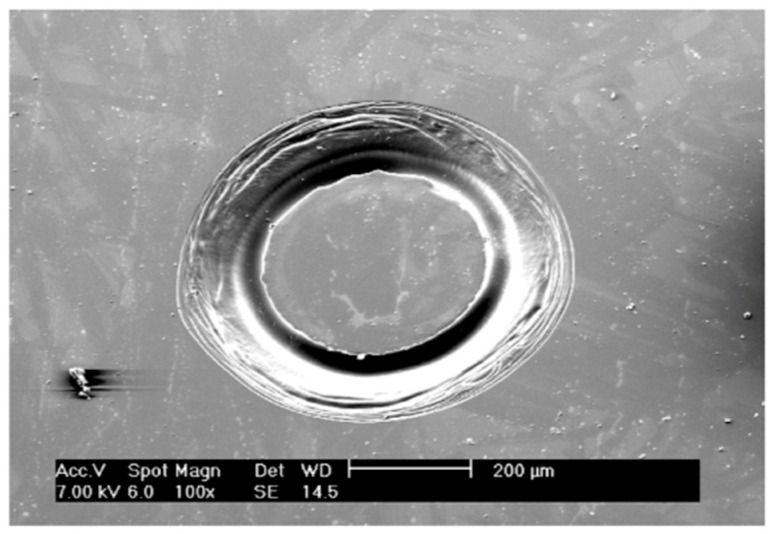
Crater created by a cylindrical tool electrode. Machined with 35-V electrode voltage and 2-s machining time [26].

The formation of this protrusion structure may be explained by two aspects. On the one hand, the discharge is more difficult to generate at the center of the tool electrode that is flat-bottomed. Specifically, Jiang et al. [26,27] conducted a finite element analysis on the current density of the tool electrode with this shape, as shown in Figure 7. Compared to the center of the cylindrical tool electrode, the current density near the edge of the contour is stronger. Therefore, if geometric defects are not considered, sparks tend to generate along the edge of the tool electrode, and it is more difficult to generate near the geometric center of the bottom, so the material in this part is more difficult to remove. Figure 8 is the picture of a crater created by the ECDM process using a cylindrical tool of 0.5-mm diameter with the discharging duration of 2 s, which illustrates that material near the rim of the cylindrical tool was removed, indicating release of sparks distributed around the rim, or the “fringing effect”. On the other hand, during the ECDM, bubbles will produce at the bottom of the tool electrode. Because the solution has a high viscosity and the bubbles mainly stressed in the vertical direction, the bubbles will accumulate at the bottom of the electrode and are difficult to discharge, which hinders the electrolyte into the gap between the tool electrode and the workpiece, making this part difficult to produce electrochemical discharge [28], and the material is difficult to remove. As a result, the morphology with protrusions at the bottom is created.

It can be clearly seen from the topography on the right side of the dotted line in Figure 6 that the protrusions in the microgrooves were removed after laser machining, resulting in a smoother surface. Because ECDM preprocessing can promote the nonlinear absorption of the high-energy pulsed laser in the processing area and induce plasma generation. It can selectively remove the protrusion structures of different shapes formed in the ECDM. Besides, when the picosecond pulsed laser acts on a transparent medium, the high peak power can produce a strong nonlinear effect inside the transparent medium, and reduce the heat-affected zone effectively, which greatly improves the morphology quality.

But the drawbacks of this processing method are obvious. Although laser scanning can effectively remove the protrusion structures, the removed debris still existed at the bottom, and the surface roughness was not good. To further improve the morphology quality and composite effect, and to explore the influence of laser preprocessing on the gas film formation and material ablation in ECDM, the sequence of laser machining and ECDM was adjusted to form a laser-assisted ECDM microgroove machining method, as given in the details of the next section.

### 3.2. Laser Preprocess

In the laser preprocess experiments, the microgroove was processed by laser first, and then post-processed by ECDM. Specifically, the tool electrode with a suitable diameter was selected according to the width of the microgroove processed by laser pre-machining. In this experiment, the width W of the micro-groove was 200–240 μm, and the gas film thickness h was about 20 μm. The tool electrode diameter $d_{t}$ is:

(1) $$d_{t}=W-2h$$

therefore, a tool electrode with a diameter of 200 μm was selected.

**Figure 9:**
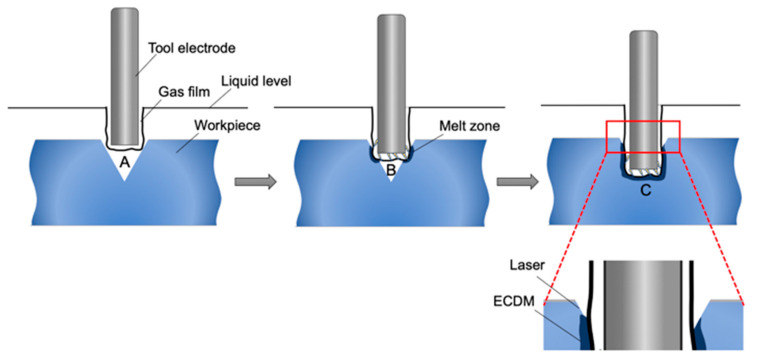
Schematic diagram of tool electrode position.

During the machining process, the relative position of the tool electrode tip and the pre-groove was monitored by connecting the micro camera to the computer to make the traces of ECDM and laser machining overlap accurately and completely. Figure 9 illustrates the change in the relative position of the tool electrode in the laser preprocessed groove during ECDM. First, the tool electrode descended to position A, where the tip of the tool electrode was slightly lower than the microgroove inlet level, but it was not in contact with the sidewall. ECDM was performed to remove burrs without forming a heat-affected zone on the edge of the microgroove. As the tool electrode continued to dive, it arrived at position B. At this time, there should be contact between the tool electrode and the sidewall of the microgroove. However, due to the discharge, this part formed a melting zone, which is further melted and eroded under the action of the electrolyte. As the tool electrode continued to dive, it finally reached position C, the bottom of the microgroove. As the ECDM proceeded, the cross-section is changing gradually from V-shaped to U-shaped, and the taper decreased significantly.

**Figure 10:**
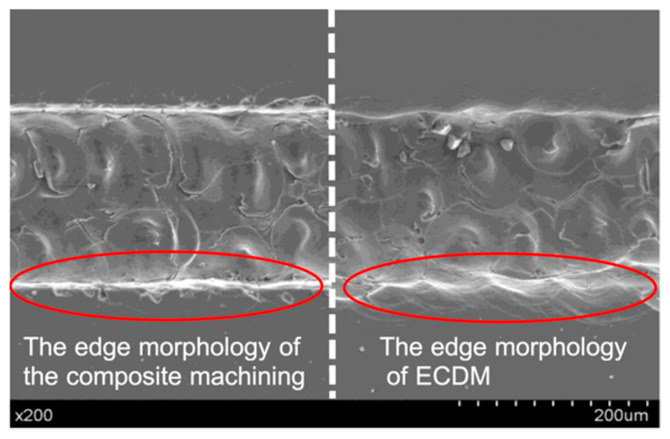
Comparison of microgrooves processed by combination method and ECDM only.

Figure 10 is a comparison diagram of the microgrooves with a width of 200 μm processed by combination method and ECDM only. Taking the dotted line in the figure as the boundary, the left side of the dotted line is combination, and the right side represents ECDM only.

It can be clearly seen that the edges of the microgroove processed by ECDM only are tortuous. In addition, the processing quality is relatively poor, and there is a wave-shaped heat-affected zone on the upper surface of the microgroove edge. This is because the molten material overflowed under the action of gas film agitation and thermal impact during the processing, and then cooled and solidified and accumulated on the edge of the microgroove, eventually forming a wave shape slightly higher than the surface of the material.

The edge of the microgroove processed by the combination method is flat, and the sidewall profile is more regular and smoother, and there is no visible wave-shaped heat-affected zone on the upper surface of the edge, showing the morphology of laser processing only.

Gas film is an important parameter which formed around the tool electrode and leads to discharge activities. The size and stability of the gas film significantly affected the material removal and accuracy of the microgrooves. A more stable gas film prepares a suitable condition for uniform discharges. The edge of the microgrooves presents the morphology of laser machining instead of ECDM, showing the microgrooves formed by laser preprocessing can reduce the thickness of the gas film, shorten the distance of electrical discharge erosion, and achieve better quality and less overcutting. This was because of the lack of material produced by laser processing increased the thickness of the liquid layer between the edge of the microgroove and the discharge area of the tool electrode, which increased the stability of the gas film. At the same time, the processed microgrooves squeezed the gas film on the tip of the tool electrode, which had a restrictive and standard effect on the ECDM energy. The dual action improved the localization of ECDM, reduced the amount of overcutting, and greatly improved the machining accuracy. Therefore, the edge of the laser preprocessed microgroove was not affected by the discharge, and still presents a neat edge of the preprocessed laser.

**Figure 11:**
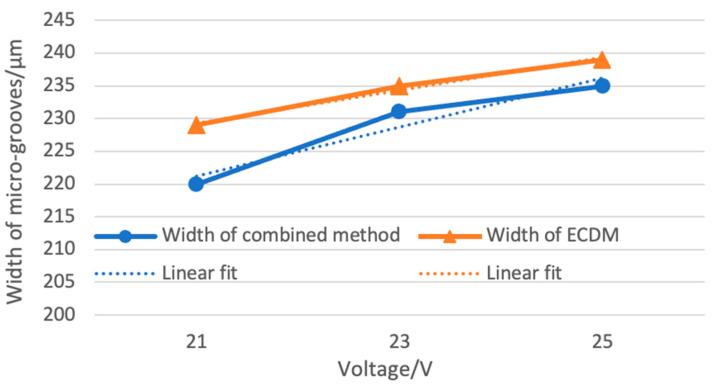
Comparison of width processed by combination method and ECDM only.

While the bottom surface presents the morphological characteristics of ECDM instead of laser machining. Since the microgrooves processed by the laser show a certain taper, the width of the microgrooves become narrower as the depth of the microgrooves increases. When the distance between the material and the tool electrode is less than the critical distance of electric discharge erosion (Figure 9, positions B and C), the material at that place will be eroded by ECDM. And since the final position of the tool electrode in the bottom of the groove reaches the effective processing range, the depth of ECDM is deeper than laser, so the bottom surface presents the morphological characteristics of ECDM instead of laser machining. As shown in the enlarged detail of Figure 9, laser-assisted ECDM can keep the laser processing morphology on the upper edge of the microgroove, and ECDM only affects the middle and lower regions. Therefore, the experiment can obtain high-quality microgrooves, which can have flat edges and small tapers. As shown in Figure 11, the width of the microgrooves of the combined machining is generally smaller than that of the ECDM. This study used different voltage parameters to verify that the combined method can improve the overcut phenomenon caused by ECDM, thereby improving the processing quality.

In summary, the microgroove first processed by laser, and then a tool electrode with an appropriate diameter was selected for ECDM according to the width of the groove. The combination of the two methods can effectively improve the edge quality of the microgrooves to obtain a more straight and neat edge morphology with less overcutting. At the same time, the taper of the microgroove can be effectively reduced, and the cross section is closer to a U-shape.

## 4. Conclusions

This study explored ECDM and laser processing of quartz, and compared the morphological differences between single processing methods and combined processing methods. The experimental results are summarized:

1. As a post-processing after ECDM, laser processing can effectively and selectively remove the protrusions at the bottom of the microgrooves. Because of the “cold processing” feature of the picosecond laser, there is no obvious heat-affected zone. Thus, the experiment obtained a more regular bottom structure of the microgrooves.
2. In the laser preprocessing, the pre-etched microgrooves can reduce the thickness of the gas film, shorten the distance of the electrical discharge erosion, and achieve better quality. The results show the bottom of the microgroove presented an ECDM morphology, while the upper surface of the edge presented a laser morphology and there is no visible wave-shaped heat-affected zone. The combined processing method can improve the overcut phenomenon caused by ECDM.
3. After the post-processing by ECDM, the V-shaped microgrooves gradually transform to the U-shape, and the taper of the microgrooves is reduced.

## Figures and Tables

**Table 1:** Parameters for ECDM.

Factors	Parameters
Tool electrode diameter (μm)	200
Voltage (V)	25
Peak current (A)	0.3
Duty factors (%)	50
Frequency (Hz)	1000
Rotating speed (RPM)	3600
Feed speed (μm/s)	10

**Table 2:** Parameters for laser machining.

Factors	Parameters
Layer	10
Number of elements	20
Scanning speed (mm/s)	300
Power (W)	12
Frequency (Hz)	3 × 10^5^

## Data Availability

Data available on request due to restrictions e.g., privacy or ethical. The data presented in this study are available on request from the corresponding author. The data are not publicly available due to it was not agreed by all co-authors.
